# GTSE1 promotes cell migration and invasion by regulating EMT in hepatocellular carcinoma and is associated with poor prognosis

**DOI:** 10.1038/s41598-017-05311-2

**Published:** 2017-07-11

**Authors:** Xiaojuan Wu, Hongbo Wang, Yifan Lian, Lubiao Chen, Lin Gu, Jialiang Wang, Yanlin Huang, Meihai Deng, Zhiliang Gao, Yuehua Huang

**Affiliations:** 1 Department of Infectious Diseases, The Third Affiliated Hospital of Sun Yat-sen University, Guangzhou, 510630 China; 2 Guangdong Provincial Key Laboratory of Liver Disease Research, The Third Affiliated Hospital of Sun Yat-sen University, Guangzhou, 510630 China; 3 Department of Hepatobiliary Surgery, The Third Affiliated Hospital of Sun Yat-sen University, Guangzhou, 510630 China

## Abstract

G2 and S phase-expressed-1 (GTSE1) regulates G1/S cell cycle transition. It was recently reported to be overexpressed in certain human cancers, but its significance and mechanism(s) in hepatocellular carcinoma (HCC) remain unknown. Here, we showed preferential GTSE1 upregulation in human HCC tissues and cell lines that positively correlated with Ki67. GTSE1 knockdown by short hairpin RNA resulted in deficient colony-forming ability and depleted capabilities of HCC cells to migrate and invade. Conversely, exogenous GTSE1 overexpression enhanced colony formation and stimulated HCC cell migration and invasion. Furthermore, GTSE1 silencing was associated with the downregulation of N-cadherin, β-catenin, and Snail, whereas GTSE1 overexpression caused the opposite effects. GTSE1 upregulated Snail via both transcription and protein degradation pathways. Additionally, GTSE1 modulated the sensitivity of HCC to 5-fluorouracil therapy. High GTSE1 correlates with chemo-resistance, while low GTSE1 increases drug sensitivity. Kaplan-Meier survival analysis indicated that high GTSE1 levels were significantly associated with poor overall survival. In conclusion, high expression of GTSE1 is commonly noted in HCC and is closely correlated with migration and invasion by epithelial-to-mesenchymal transition (EMT) modulation. Activated GTSE1 significantly interferes with chemotherapy efficacy and influences the probability of survival of patients with HCC. GTSE1 may thus represent a promising molecular target.

## Introduction

Hepatocellular carcinoma (HCC) is the third leading cause of cancer-related death worldwide, leading to the deaths of approximately 700,000 people per year^1^. Current treatment options for HCC are limited and generally ineffective. The only curative treatment is surgical resection or liver transplantation. However, most patients are ineligible for surgery due to the late stage of the disease at the time of diagnosis^2^. A better understanding of the molecular mechanisms underlying liver carcinogenesis and further studies of HCC oncogenes may lead to advances in the identification of novel molecular markers of HCC progression and the development of new diagnostic and therapeutic strategies.

Deregulation of cell cycle regulators is one of the major factors contributing to HCC development and tumour progression^3^. Numerous studies indicate that abolishing G1 arrest and/or stimulating G1/S phase transition in the cell cycle facilitate the unrestrained growth of unstable cells, precancerous cells, or cancer cells and are associated with hepatocarcinogenesis and HCC progression^4, 5^. In addition to genes that control the G1 or S phases, G2 and S phase-expressed-1 (GTSE1), which is expressed specifically during the G2 and S phases in the cell cycle, was recently reported to negatively regulate p53 by stimulating the cytoplasmic localization of p53 and regulating the stability of p21^6–10^. Previous studies have shown that GTSE1 is involved in human cancers, including the inhibition of apoptotic signalling to confer cisplatin resistance in gastric cancer cells^11^ and overexpression in lung and liver cancer tissues^12, 13^. However, its function in HCC progression and the underlying molecular mechanisms remain obscure.

In the present study, we demonstrated that GTSE1 was significantly upregulated in human HCC, and this elevated expression of GTSE1 suggested a poor survival. Further investigations indicated that GTSE1 functioned in promoting migration and invasion by the disruption of epithelial-to-mesenchymal transition (EMT). In addition, silencing GTSE1 enhanced the effects of 5-FU in HCC. We evaluated the role of GTSE1 as a prognostic marker and a therapeutic molecular target in HCC.

## Results

### GTSE1 is frequently upregulated in HCC

**Figure 1:**
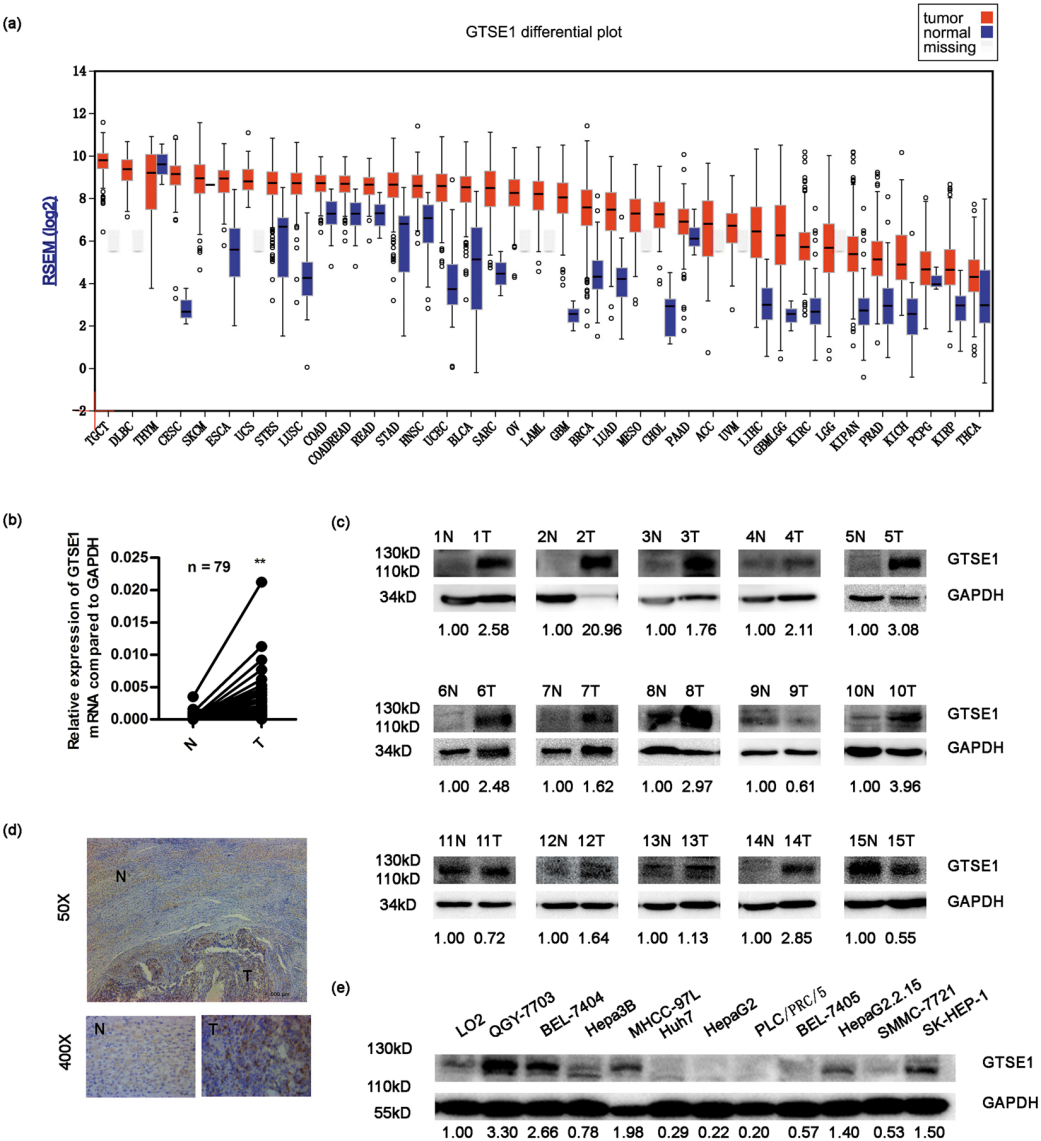
GTSE1 is frequently upregulated in HCC. (**a**) The mRNA levels of GTSE1 in global human cancer tissues (red) and non-tumour tissues (blue) were analysed based on the TCGA database. ACC: Adrenocortical carcinoma; BLCA: Bladder urothelial carcinoma; BRCA: Breast invasive carcinoma; CESC: Cervical squamous cell carcinoma and endocervical adenocarcinoma; CHOL: Cholangiocarcinoma; COAD: Colon adenocarcinoma; COADREAD: Colon and rectum adenocarcinoma; DLBC: Diffuse large B-cell lymphoma; ESCA: Esophageal carcinoma; GBM: Glioblastoma multiforme; GBMLGG: Glioblastoma multiforme and brain lower grade glioma (GBM + LGG); HNSC: Head and neck squamous cell carcinoma; KILH: Kidney chromophobe; KIPAN: Pan-kidney cohort (KICH + KIRC + KIRP); KIRP: Kidney renal papillary cell carcinoma; KIRC: Kidney renal clear cell carcinoma; LAML: Acute myeloid leukemia; LGG: Brain lower grade glioma; LIHC: Liver hepatocellular carcinoma; LUAD: Lung adenocarcinoma; LUSC: Lung squamous cell carcinoma; MESO: Mesothelioma; OV: Ovarian serous cystadenocarcinoma; PAAD: Pancreatic adenocarcinoma; PCPG: Pheochromocytoma and paraganglioma; PRAD: Prostate adenocarcinoma; READ: Rectum adenocarcinoma; SARC: Sarcoma; SKCM: Skin Cutaneous melanoma; STAD: Stomach adenocarcinoma; STES: Stomach and esophageal carcinoma; TGCT: Testicular germ cell tumors; THCA: Thyroid carcinoma; THYM: Thymoma; UCEC: Uterine corpus endometrial carcinoma; USC: Uterine carcinosarcoma; UVM: Uveal melanoma. (**b**) The mRNA levels of GTSE1 from 79 patients were tested by quantitative PCR using paired T-test. GAPDH was used as an internal control. (**c**) The protein levels of GTSE1 in HCC tissues and matched non-tumour tissues from 15 patients with HCC were determined by western blotting assay. Relative fold changes of T compared with N are below. (**d**) Immunohistochemistry analysis of GTSE1 distribution in the human liver tissues. (**e**) GTSE1 expression in primary HCC cell lines. Relative fold changes of HCC cell lines compared with LO2 are below. Original data are presented in Supplementary Fig. 2. N: adjacent non-tumour tissue; T: tumour tissue. Statistically significant difference: *P < 0.05, **P < 0.01.

To investigate the differential expression of GTSE1 in different human tumours, we analysed the mRNA expression profiles of various tumour tissues and compared them with those of non-tumour tissues using the TCGA data analysis website (http://firebrowse.org). Thirty-seven types of human tumours were included, of which 9 types were excluded due to missing normal tissue data, leaving 28 types of cancer for analysis. The majority (27/28, 96.4%) of cancers, including HCC, showed increased levels of GTSE1 in tumour tissues compared with non-tumour tissues. The GTSE1 level was approximately 100-fold higher in cancer tissues than in non-cancerous tissues (Fig. 1a). To clarify GTSE1 expression in HCC tissues *in situ*, GTSE1 mRNA and protein levels were evaluated. As shown in Fig. 1b and c, GTSE1 mRNA expression (75/79, 94.9%) and GTSE1 protein levels (12/15, 80.0%) were significantly upregulated in HCC tissues compared with relative adjacent tissues. The high expression of GTSE1 in fresh HCC tissues *in situ* was further confirmed by immunohistochemistry (IHC, Fig. 1d). The GTSE1 protein was predominantly expressed in the nuclei and plasma of the HCC tumour regions (T), whereas GTSE1 was only occasionally expressed in the liver cells of the adjacent non-cancerous tissues (N). To investigate GTSE1 expression in HCC cell lines, GTSE1 protein levels were analysed by western blot analysis. Compared with the immortalized human liver cell line LO2, the QGY-7703, BEL-7404, Hepa3B, MHCC-97L, HepaG2.2.15, and SK-HEP-1 cell lines showed elevated protein expression levels of GTSE1 (Fig. 1e). Taken together, these results demonstrated that GTSE1 expression was increased in HCC tumour tissues and implied that the upregulation of GTSE1 in HCC might play a considerable role in tumour development.

### GTSE1 accelerates tumour progression in HCC

**Figure 2:**
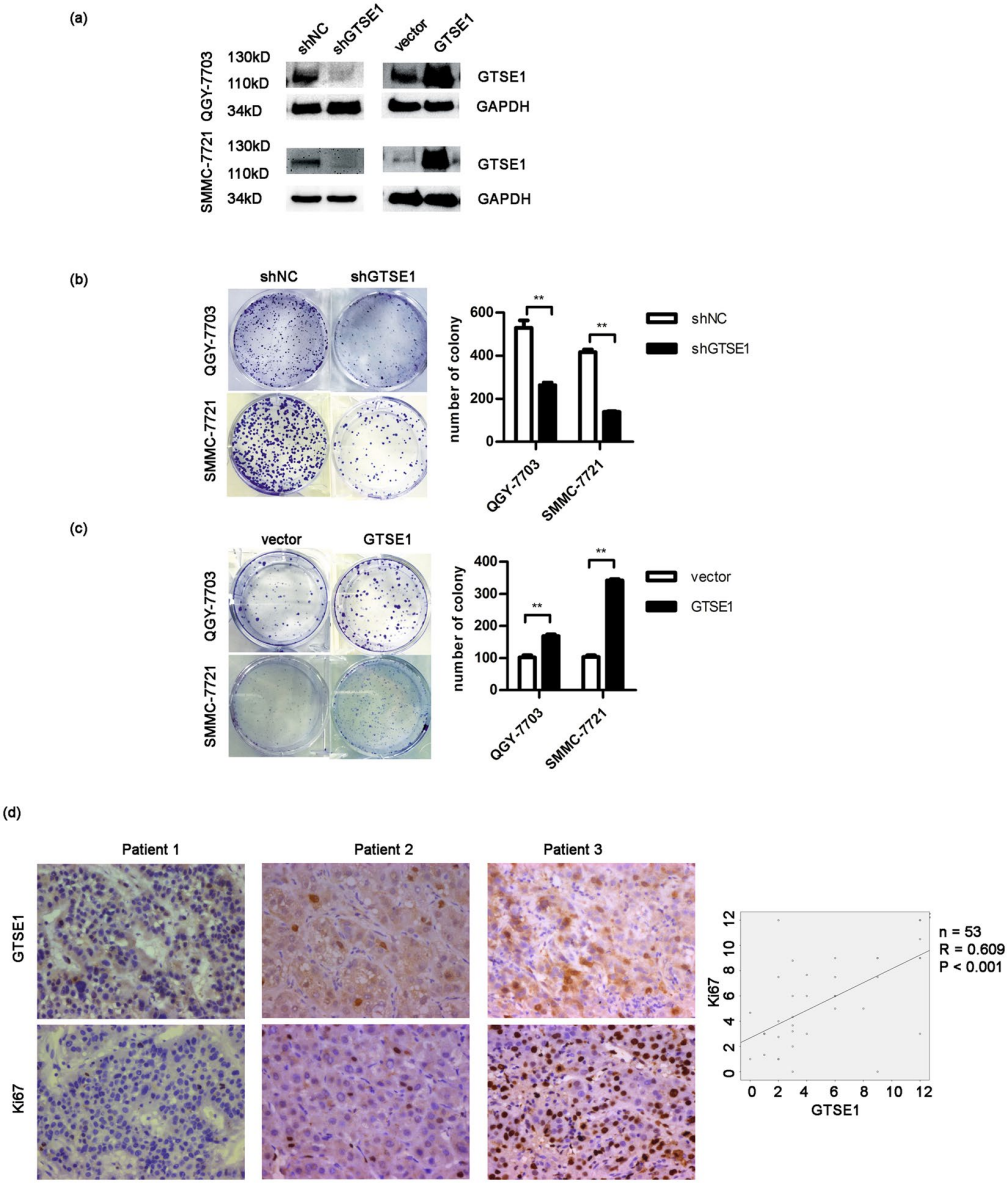
GTSE1 enhances cancer progression in HCC. (**a**) Expression of GTSE1 in QGY-7703 and SMMC-7721 cells stably expressing shGTSE1 or GTSE1 and controls. Original data are presented in Supplementary Fig. 3. (**b**) Colony formation assays using QGY-7703 and SMMC-7721 cells transfected with GTSE1-shRNA (shGTSE1) or scramble (shNC). (**c**) Colony formation assays using QGY-7703 and SMMC-7721 cells transfected with GTSE1-GFP (GTSE1) or control-GFP (vector). (**d**) Representative expression levels of GTSE1 and Ki67 in HCC tissues by immunohistochemistry. Bivariate correlation analysis was employed to analyse the correlation between GTSE1 and Ki67.

To address the cellular mechanisms of GTSE1 responsible for tumour progression, we established stable hepatocellular cell lines (QGY-7703, SMMC-7721) in which GTSE1 was suppressed via a shRNA expressing vector or overexpressed using a GTSE1 expressing vector. Protein levels were determined to confirm that GTSE1 silencing and overexpressing cell lines were successfully constructed (Fig. 2a). Firstly, we performed foci formation assays as described. The number of colonies was significantly reduced in GTSE1-suppressing cells (QGY-7703: 529.67 ± 59.53 vs. 262.67 ± 21.385, P = 0.002; SMMC-7721: 416.33 ± 21.962 vs. 139.00 ± 5.292, P = 0.001; Fig. 2b), and significantly increased in GTSE1-expressing cells (QGY-7703: 102.33 ± 11.68 vs. 168.33 ± 9.29, P = 0.002; SMMC-7721: 103.67 ± 9.29 vs. 342.33 ± 7.024, P < 0.001; Fig. 2c). Next, we examined the relationship between GTSE1 and Ki67 by investigating their expression simultaneously in 53 fresh HCC tissues by IHC. As shown in Fig. 2d, we observed a significant positive correlation between GTSE1 and Ki67 (R = 0.609, P < 0.001). Taken together, these results implied that GTSE1 played a vital role in HCC progression.

### GTSE1 facilitates migration and invasion *in vitro*

**Figure 3:**
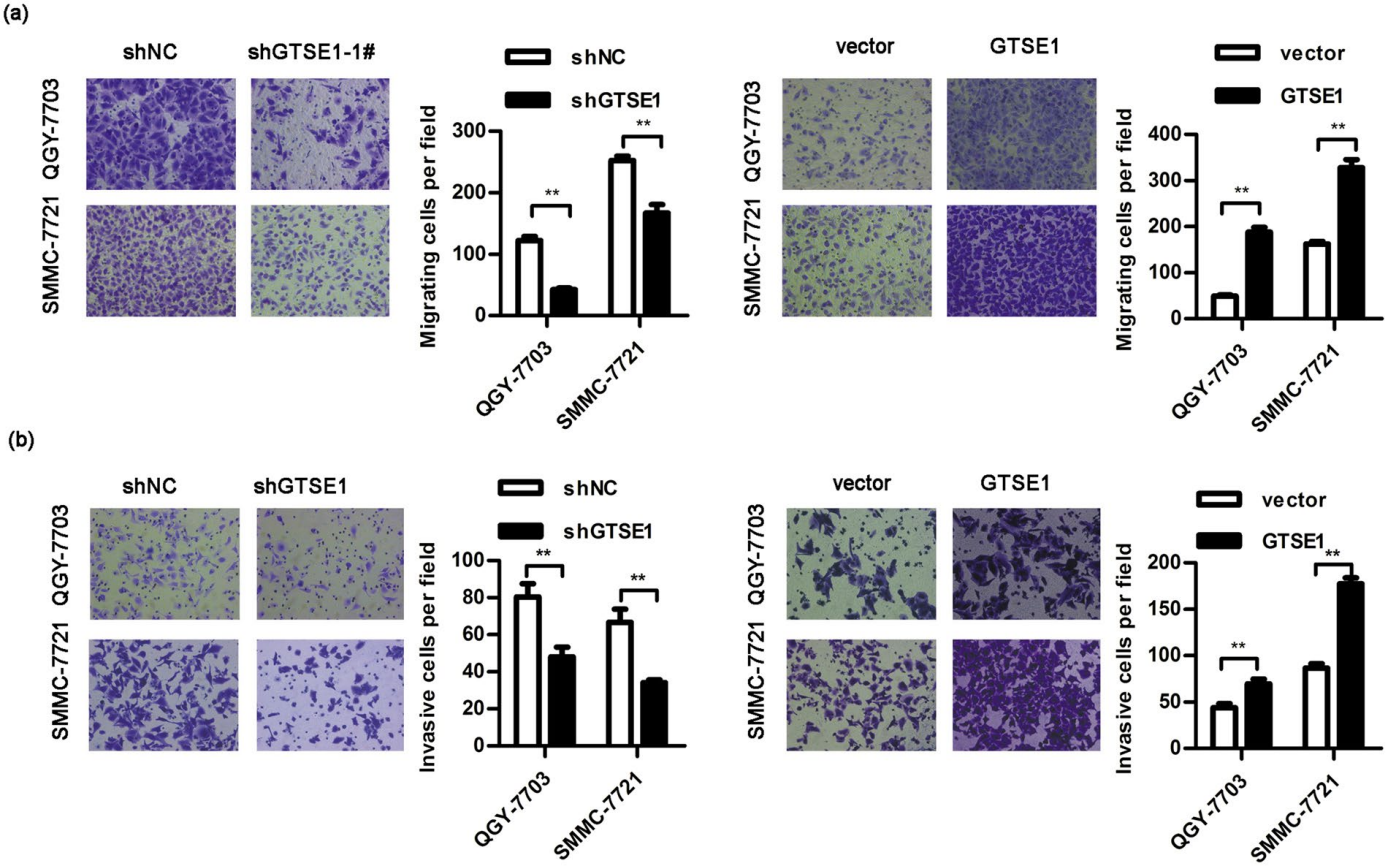
GTSE1 facilitates migration and invasion *in vitro*. (**a**) The migration assay using QGY-7703 and SMMC-7721 transfected with shGTSE1 or GTSE1 or control. (**b**) The invasion potential of QGY-7703 and SMMC-7721 transfected with shGTSE1 or GTSE1 or control. Statistical method: independent *t*-test.

Our previous results showed that GTSE1 was upregulated in human HCC and influenced tumour progression. We further identified its role in metastasis. Transwell assays were performed to explore the effects of GTSE1 on cellular motility. The inhibition of endogenous GTSE1 dramatically decreased the number of cancer cells during migration (QGY-7703: 122.38 ± 18.53 vs. 42.25 ± 7.15, P < 0.001; SMMC-7721: 252.25 ± 21.18 vs. 166.88 ± 39.15, P < 0.001; Fig. 3a) or invasion (QGY-7703: 90.50 ± 19.93 vs. 58.13 ± 14.86, P = 0.002; SMMC-7721: 66.63 ± 20.18 vs. 34.12 ± 4.26, P = 0.002; Fig. 3b) through transwell membranes. Conversely, ectopic expression of GTSE1 significantly increased cellular migration (QGY-7703: 42.00 ± 8.00 vs. 175.75 ± 35.31, P < 0.001; SMMC-7721: 162.50 ± 15.15 vs. 328.13 ± 49.65, P < 0.001; Fig. 3a) and invasion (QGY-7703: 86.75 ± 12.85 vs. 177.57 ± 17.05, P < 0.001; SMMC-7721: 78.50 ± 17.62 vs. 137.63 ± 27.38, P < 0.001; Fig. 3b). Thus, GTSE1 is considered as a stimulator of tumour migration and invasion.

### GTSE1 promotes EMT and modulates sensitivity to 5-fluorouracil therapy in HCC

**Figure 4:**
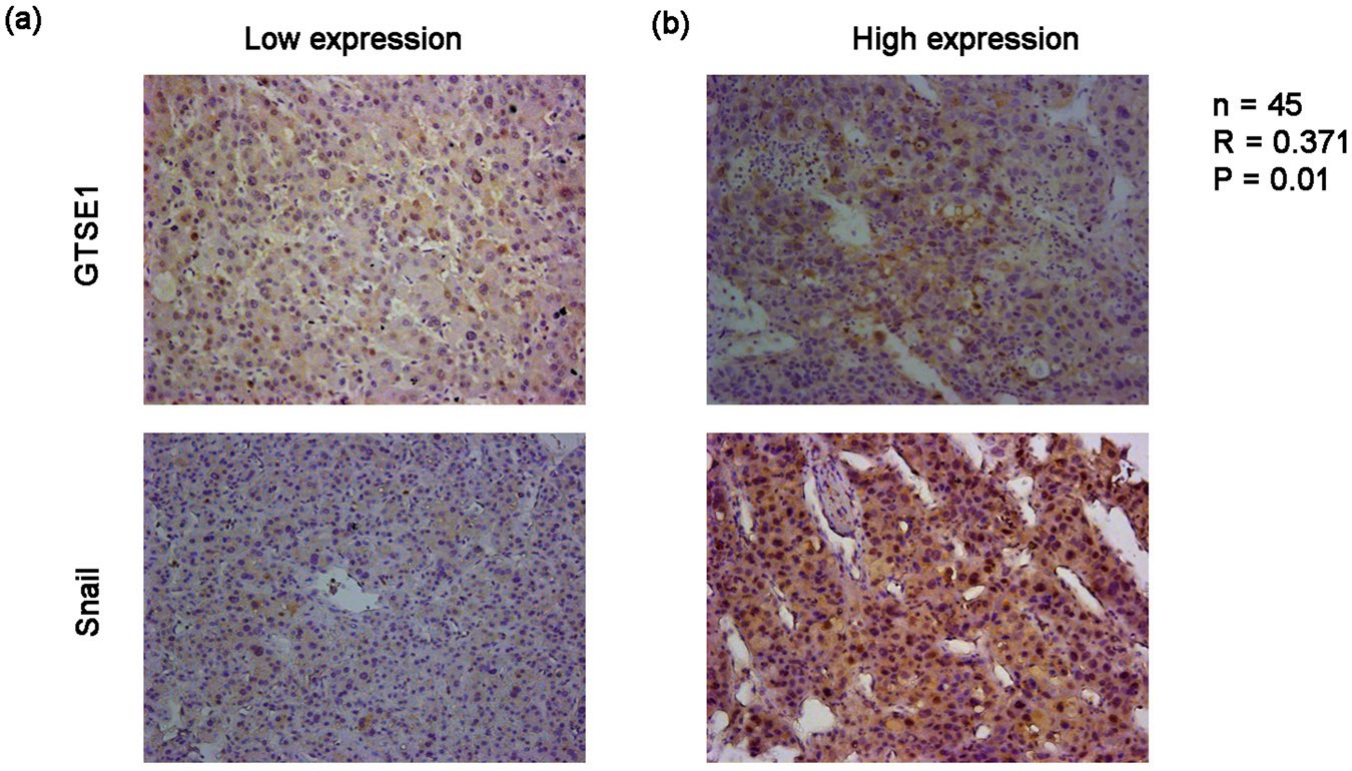
GTSE1 positively correlates with Snail *in situ*. IHC analysis of GTSE1 and Snail levels in HCC tissues with low (**a**) or high (**b**) expression.

**Figure 5:**
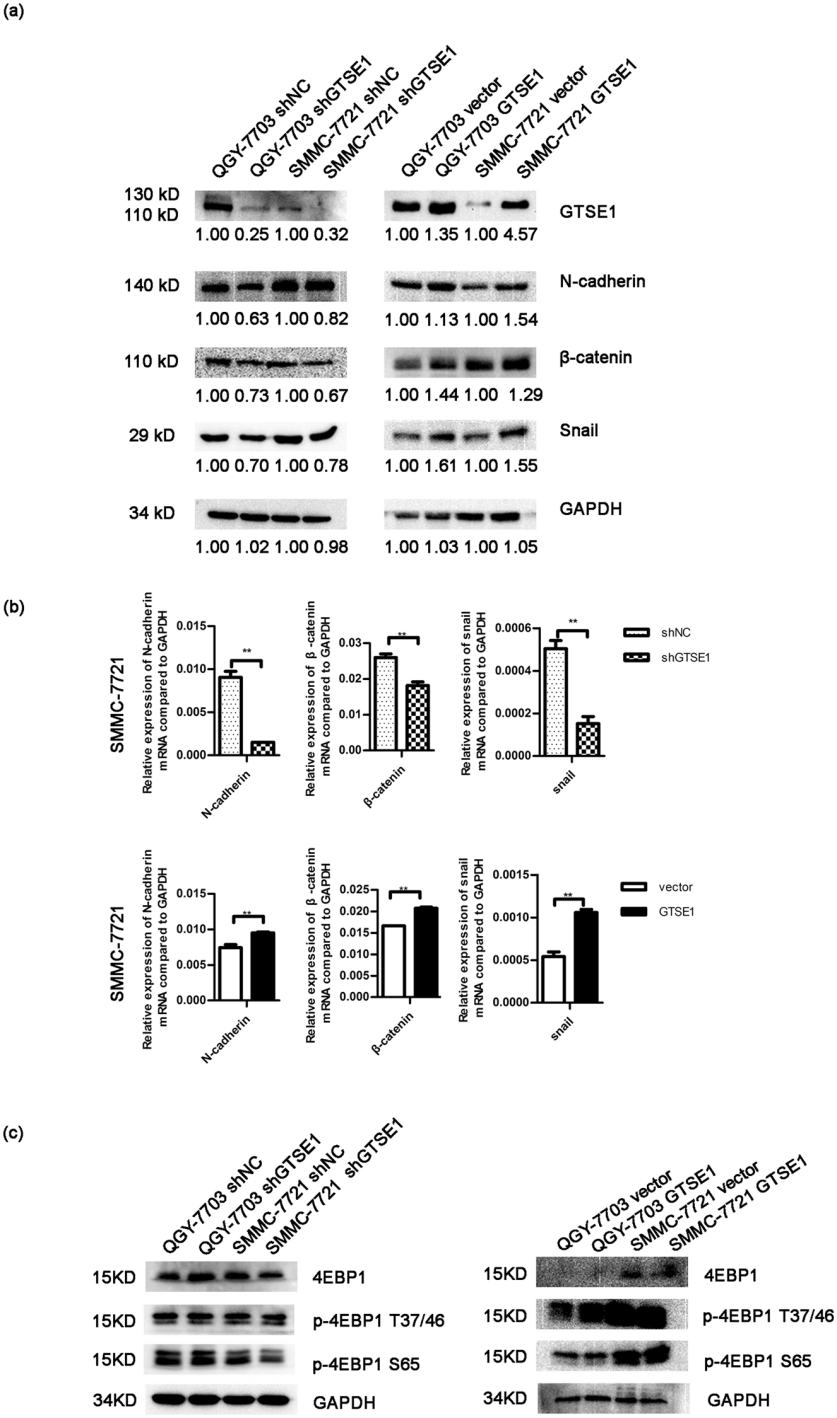
GTSE1 regulates EMT *in vitro*. (**a**) The protein levels of GTSE1, N-cadherin, β-catenin, and Snail were determined by western blotting in QGY-7703 and SMMC-7721 cells stably transfected with shGTSE1 or GTSE1 or control. Relative protein fold changes compared with the control are marked below. Original data are presented in Supplementary Fig. 4. (**b**) The mRNA expression levels of N-cadherin, β-catenin, and Snail were detected by qPCR in SMMC-7721. (**c**) The protein levels of 4EBP1, p-4EBP1 (T37/46), and p-4EBP1 (S65) were tested by western blotting in QGY-7703 and SMMC-7721. Original data are presented in Supplementary Fig. 5.

**Figure 6:**
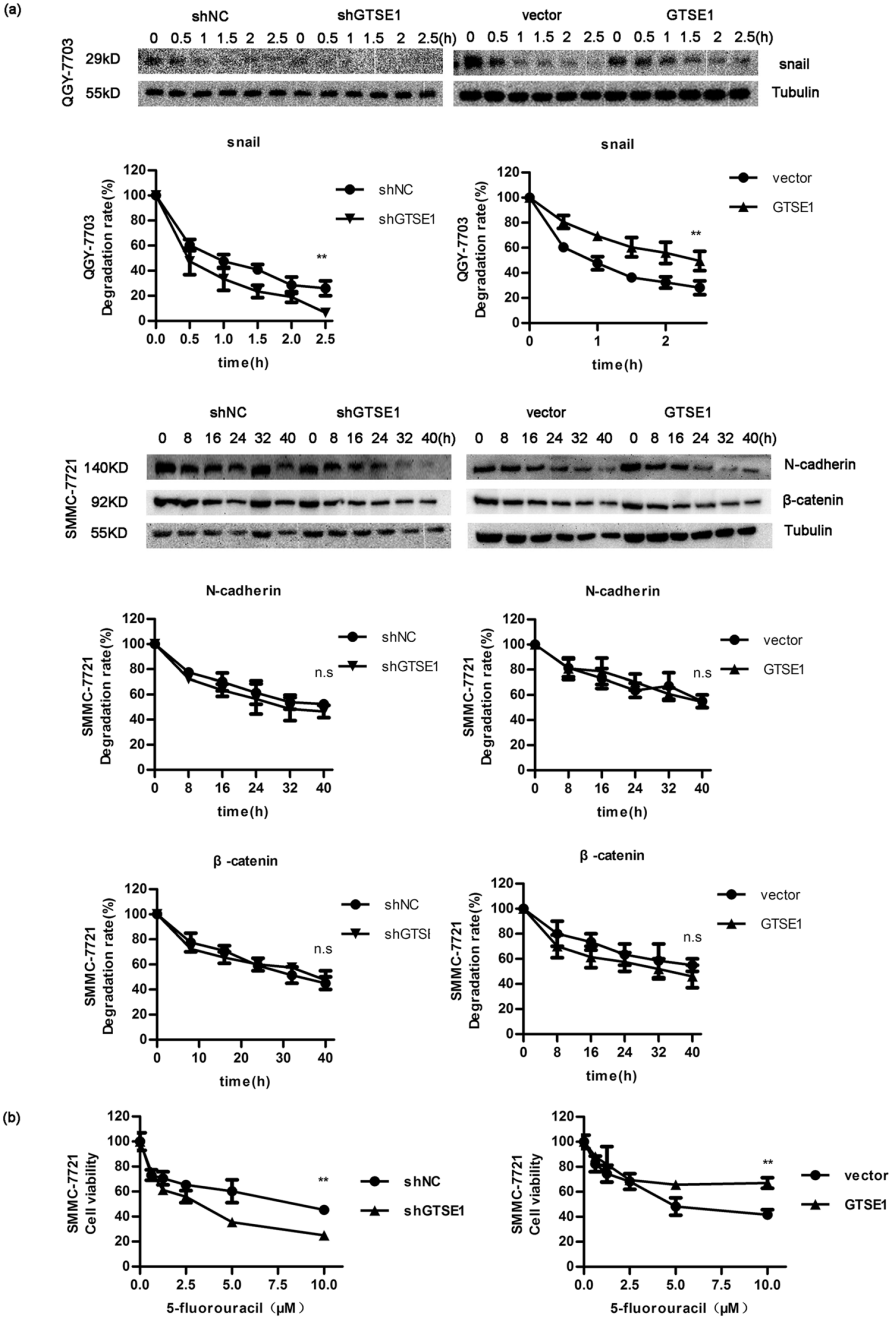
GTSE1 affects the degradation rate of Snail and modulates sensitivity to 5-FU therapy. (**a**) The protein degradation rates of N-cadherin, β-catenin, and Snail after being treated with CHX. Original data are presented in Supplementary Fig. 6. (**b**) Cell viability was tested by CCK-8.

N-cadherin, β-catenin, and Snail are well known as specific markers of EMT. We first explored the expression of Snail and GTSE1 in tissues from 45 cases of HCC by IHC. The GTSE1 level was significantly correlated with the level of Snail (R = 0.371, P = 0.011, Fig. 4). Then, we explored the variation of these proteins affected by GTSE1 *in vitro*. Compared with the control group, GTSE1 silencing and GTSE1 overexpressing cells showed decreased and increased expression of N-cadherin, β-catenin, and Snail, respectively (Fig. 5a), suggesting that GTSE1 was a driving force of EMT in HCC. This regulation of Snail by GTSE1 was due to both transcription and protein degradation mechanisms (Figs 5b and 6a). In addition, N-cadherin and β-catenin were modulated by GTSE1 via a transcriptional pathway (Figs 4b and 6a). However, GTSE1 did not appear to affect the translation level of these markers (Fig. 5c).

Cell death resistance is a key feature of EMT^14^. As GTSE1 was reported to serve as a regulator of gastric cancer against cisplatin therapy^11^, we then investigated whether GTSE1 could affect the anti-tumour effects of 5-fluorouracil (5-FU) in HCC. Different concentrations of 5-FU (0, 0.625, 1.25, 2.5, 5, and 10 µM) were applied to treat GTSE1 silencing or overexpressing cells. When GTSE1 was inhibited, cell viability declined markedly (Fig. 6b). When cells overexpressed GTSE1, more cells survived after 5-FU treatment (Fig. 6b). These results showed that GTSE1 provokes chemo-resistance to 5-FU therapy, and silencing GTSE1 enhanced the anti-tumour effects of 5-FU in HCC. GTSE1 proved able to affect the sensitivity of cells to chemotherapeutic drugs.

### High GTSE1 expression correlates with a poor prognosis

**Figure 7:**
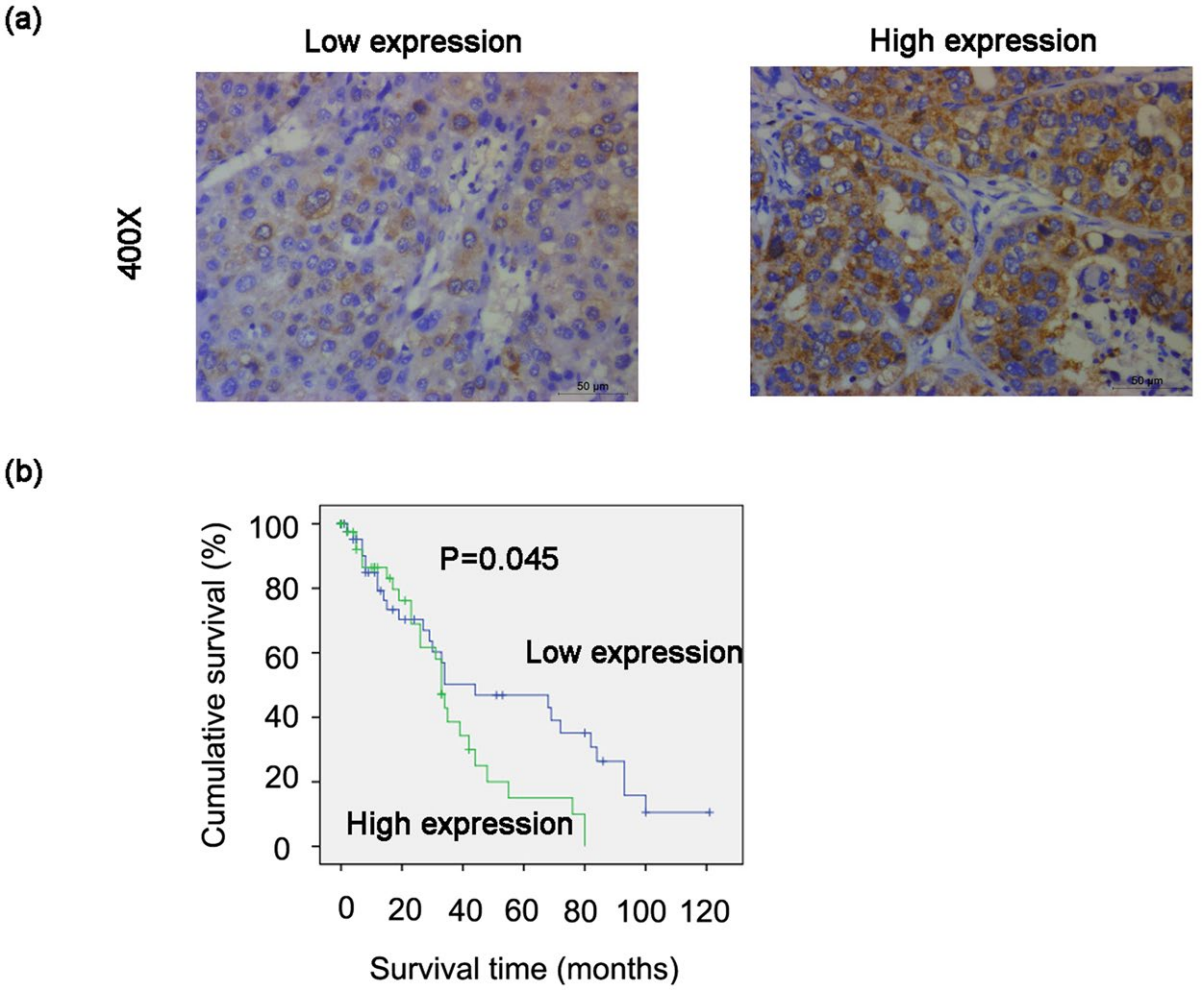
High expression of GTSE1 in HCC predicts a poor prognosis. (**a**,**b**) Kaplan-Meier analysis for the expression of GTSE1.

We finally investigated the role of GTSE1 in the outcome of human HCC. Immunohistochemical analysis using an anti-GTSE1 polyclonal antibody was performed in 89 patients with primary HCC. All tissues were received from surgically resected primary tumours. Of the 89 cases, 83 cases (93.25%) revealed positive GTSE1 staining, while 6 cases (6.74%) revealed negative GTSE1 staining. Based on staining extent and intensity, a total of 42 (47.19%) were categorized as having high GTSE1 expression, and 47 (52.81%) were categorized as having low expression (Fig. 7a). We then examined the association of GTSE1 expression with clinical outcomes. Statistical analysis using the Kaplan-Meier method revealed a striking association between GTSE1 expression and tumour-specific 10-year overall survival (OS). The cumulative probability of OS at 5 years was 0.150 ± 0.088 and 0.449 ± 0.076, with higher or lower GTSE1 expression in HCC, respectively (Fig. 7b). As expected, GTSE1 levels significantly predicted overall survival in human HCC. These data indicated a crucial role of GTSE1 in the prediction of clinical outcome in HCC. Next, we found the relationship between GTSE1 expression and clinicopathological features. A Chi-square analysis showed that patients with higher GTSE1 expression were more likely to have multiple tumours and to have higher alpha-fetoprotein (AFP) levels, whereas patients with lower GTSE1 expression were prone to having a single tumour and lower AFP levels (Table 1). Taken together, these findings implied that GTSE1 played a vital role in HCC progression and might represent a prognostic biomarker for patients with HCC.

**Table 1:** Correlation between GTSE1 and clinicopathological features in patients with HCC.

		GTSE1 (n = 89)		P value
		Low expression (n = 47)	High expression (n = 42)	
1. Age (year)	≤50	30	19	0.078
	>50	17	23	
2. Gender	Male	37	34	0.794
	Female	10	8	
3. Size of tumour	≤5 cm	19	19	0.710
	>5 cm	27	23	
4. Number of tumours	1	46	29	<0.001**
	≥2	1	12	
5. Histological differentiation	High	2	4	0.257
	Medium	36	25	
	Low	8	11	
6. Tumour thrombus	No	37	38	0.714
	Yes	3	5	
7. Lymphnode metastasis	No	44	34	0.291
	Yes	3	6	
8. HBV DNA	≤1e5	38	23	0.036*
	>1e5	8	14	
9. Tumour relapse	No	30	30	0.631
	Yes	15	12	
10. AFP (ng/mL)	≤500	27	16	0.016*
	>500	4	11	

AFP, alpha-fetoprotein; *P < 0.05, **P < 0.01.

## Discussion

HCC is characterized by heterogeneous malignancy, multiple genetic and epigenetic alterations, and complex molecular signalling pathways. Current treatment limitations have led to an active search for novel genes that can implicate the malignant transformation of hepatocytes and tumour progression. Here, we reveal a novel role of GTSE1 in the clinical outcomes of HCC. We used human HCC samples and cell lines to address the cellular and molecular mechanisms by which GTSE1 promotes hepatic malignant transformation and tumour progression. The increase in GTSE1 expression was associated with poor HCC prognosis, suggesting that this gene product may function as an oncogene and may play a pivotal role in tumour progression and metastasis. Particularly, we found that GTSE1, which is key in cell-cell adhesion via the transcription and/or protein degradation pathways, enhanced EMT. GTSE1 also suppressed the sensitivity of HCC cells to chemotherapeutic drugs. Thus, this study presents a key role for GTSE1 in the growth and progression of liver cancer.

The major effect of GTSE1 in clone formation in HCC cells and the direct association with rapid proliferation in HCC tissues (high numbers of Ki67-positive cells) highlighted its important role in human HCC progression. Published studies have demonstrated that GTSE1 may serve as a negative regulator of p53^6, 8^. GTSE1 downregulates p53 by its translocation from the nucleus to the cytoplasm for degradation^9^. Thus, G2 arrest can be suppressed when responding to G2 DNA damage. This allows unstable or tumour cells to resist apoptosis and transit through the G2/M checkpoint, finally leading to tumour formation and progression. These findings consolidate previous observations that the abrogation of cell cycle checkpoints by the dysregulation of cell cycle regulators promotes cell cycle progression and cellular growth involved in hepatocarcinogenesis^4^.

Cell migration is a crucial process when cancer cells become metastatic^15^. The regulatory pathways controlling cell migration depend on the microtubule cytoskeleton and its related proteins^16, 17^. GTSE1 was recently shown to be a microtubule-associated protein^18^. It is reported to suppress mitotic centromere-associated kinesin (MCAK) to stabilize microtubules. The interaction between GTSE1 and MCAK results in chromosome segregation defects and induces chromosomal instability, eventually influencing tumourigenesis^18^. In a study on breast cancer, GTSE1 protein levels were shown to determine the migratory capacity of non-transformed breast cancer cell lines^19^. Increased GTSE1 expression correlates with invasive potential, tumour stage, and time to distant metastasis^12, 19^. Consistent with this study, we showed that the migration and invasion of HCC cells were dependent on the levels of GTSE1. The ectopic expression of GTSE1 stimulated cellular migration and invasion, while the suppression of GTSE1 reduced the cellular abilities of migration and invasion. These data indicated that the upregulation of GTSE1 could be associated with increased metastatic potential, which was consistent with a similar study by Guo *et al*.^12^. Our study utilized both overexpression models and silencing models with a different small hairpin RNA sequence from that used in the study by Guo *et al*.^12^. Instead of focusing on cell proliferation and the role of MMP2/9 in HCC metastasis, we paid more attention to the EMT mechanism (Figs 5 and 6), and we started to explore the impact of HBV infection on GTSE1 (Supplemental Fig. S1).

Increased abilities of cellular migration and invasion, as well as resistance to apoptosis, are the significant characteristics of EMT^20, 21^. Snail, N-cadherin, and β-catenin are well-known markers of EMT. In epithelial carcinomas, EMT facilitates tumour cells to depart from the primary tumour and to invade the local tumour or blood vessels. When tumour cells acquire mesenchymal-cell markers (for example, Snail and N-cadherin), they are prone to migration and invasion^21–23^. As expected, our data indicated that the overexpression of GTSE1 in HCC cells induced an increase in the EMT phenotype. The knockdown of GTSE1 had a significant impact on EMT, as shown by the decreased expression of Snail, N-cadherin, and β-catenin. This behaviour can be explained by a transcriptional regulator of N-cadherin and β-catenin as well as both transcriptional and post-transcriptional regulation of Snail by GTSE1. Importantly, our data indicated that GTSE1 strongly attenuated the sensitivity of HCC cells to 5-FU. The upregulation of GTSE1 was associated with a shorter survival duration in human HCC. Thus, the present results indicated that most aggressive HCC elaborates a complex genetic progression due to the role of GTSE1.

In contrast, we found that Hepatitis B virus (HBV) activated GTSE1 expression in HCC cell lines (Supplementary Fig. S1). Patients with chronic HBV infection have an approximately 100-fold increased risk of HCC development^24^. Several viral factors, including HBV genotype C, HBV DNA, and HBV core promoter mutations, have been shown to be independently associated with HBV-related cancer development and poor prognosis^25–27^. The HBV X (HBx) protein is a multifunctional regulator and has been implicated in hepatocarcinogenesis. The mutations in the coding region of the HBx gene may alter its biological functions and increase the risk of HCC^5^. The latter observation does not seem to apply to our data. When introducing the HBx gene instead of the HBV whole genome, the activation of GTSE1 was diminished. Therefore, we speculate that coding regions in the HBV genome other than the HBx gene play essential roles in the regulation of GTSE1. Further study is required to clarify the mechanisms by which HBV modulates GTSE1 and the effect on HBV-associated HCC.

The strengths of this study include its novel insights into the molecular mechanisms by which GTSE1 accelerates HCC progression and its inclusion of the 10-year follow up on the effect of GTSE1 on HCC survival. Nevertheless, some questions that remain unanswered in the current study and that merit future investigation: 1) identification of the molecular mechanisms that control GTSE1 to exert its effect on human HCC progression, and 2) further validation of the current findings in an animal model of the disease.

In conclusion, we have shown that GTSE1 promotes HCC proliferation, migration, and invasion and is associated with poor prognosis. It upregulates expression of EMT and reduces the sensitivity of HCC cells to chemotherapeutic drugs. Therefore, GTSE1 may act as a diagnostic biomarker and a potential target for HCC therapy.

## Materials and Methods

### Materials

The rabbit anti-GTSE1 antibody was purchased from Bethyl Laboratories (Montgomery, AL, USA). The rabbit monoclonal antibodies against N-cadherin, β-catenin, Snail, 4EBP1 and p-4EBP1 (T37/45, S65) were obtained from Cell Signaling Technology (Beverly, MA, USA). The mouse anti-Ki67 was from Thermo Fisher Scientific (MA5-14520, Rockford, USA).

### Patient selection and tissue preparation

Paraffin-embedded HCC specimens (n = 89) for prognostic survival analysis were obtained from the Cancer Center of Sun Yat-sen University (Guangzhou, China). Another 53 fresh HCC specimens, for comparing GTSE1 expression between HCC tissues and adjacent non-tumour tissues and for analysing the relationship between GTSE1 and Ki67/Snail, were collected from the Third Affiliated Hospital of Sun Yat-sen University (Guangzhou, China). Each patient underwent a surgical tumour resection. Tissues were cut into proper size and then fixed in 4% paraformaldehyde for IHC or stored in liquid nitrogen directly for RNA and protein extraction. The study was approved by the Research Ethics Committee of the Cancer Center of Sun Yat-sen University and the Third Affiliated Hospital of Sun Yat-sen University (Guangzhou, China). Informed consent was obtained from each patient involved in this study. Experiments with these samples were performed in accordance with the relevant regulations.

### Immunohistochemistry

Immunohistochemical staining (IHC) was performed as previously described^26^. Briefly, all paraffin-embedded HCC samples were cut into 4-μm sections and then deparaffinised and rehydrated. The sections were boiled in a citrate antigen retrieval solution (pH = 8.0) for 3 min in an electric pressure cooker for antigen retrieval. The IHC staining was performed with the DAKO Envision system (Dako, Carpinteria, CA, USA) following the manufacturer’s recommended protocols. Briefly, sections were treated with diluted primary antibody against GTSE1, Ki67 or Snail at 4 °C overnight and anti-rabbit/mouse secondary antibody at room temperature for 1 h. Signals were detected in a freshly prepared substrate solution (DAB) at room temperature for 5 min.

The evaluation of the staining result was performed by three independent pathologists and graded as described previously^24^, according to the expression extent scores (percentage of positive cells) and positive staining intensity (0 = no expression, 1 = weak intensity, 2 = moderate intensity, 3 = strong intensity). A final immunoreactivity score (IRS) was defined as the intensity score multiplied by the extent score for each sample.

### Cell culture

Human hepatocellular carcinoma cell lines (LO2, QGY-7703, BEL-7404, Hepa3B, MHCC-97L, Huh7, PLC/PRC/5, BEL-7405, HepaG2.2.15, SMMC-7721, and SK-HEP-1) were obtained from the College of Life Science, Sun Yat-sen University (Guangzhou, China). Cells were cultured in DMEM (Gibco, Carlsbad, CA, USA) containing 10% foetal bovine serum (FBS, Gibco) at 37 °C and 5% CO_2_. Cells were digested and passaged regularly.

### Reverse transcription and quantitative PCR

Total RNA was isolated from tissue specimens and HCC cell lines using TRIzol reagent (Invitrogen, Carlsbad, CA, USA) according to the manufacturer’s protocol. Total RNA (1 µg) was reverse transcribed into cDNA by the GoScript^TM^ Reverse Transcription System (Promega). Quantitative PCR (qPCR) were performed in two duplicate wells by employing SYBR Green (Promega, USA) in a Roche LightCycler 96 (Roche Applied Science, Penzberg, Germany). Specific primers were 5′-CAGGGGACGTGAACATGGATG-3′ (forward) and 5′-ATGTCCAAAGGGTCCGAAGAA-3′ (reverse) for GTSE1, 5′-ACTGCAACAAGGAATACCTCAG-3′ (forward) and 5′-GCACTGGTACTTCTTGACATCTG-3′(reverse) for Snail, 5′-AGCCAACCTTAACTGAGGAGT-3′ (forward) and 5′-GGCAAGTTGATTGGAGGGATG-3′ (reverse) for N-cadherin, 5′-AGCTTCCAGACACGCTATCAT-3′ (forward) and 5′-CGGTACAACGAGCTGTTTCTAC-3′ (reverse) for β-catenin, and 5′-GGAGCGAGATCCCTCCAAAAT-3′ (forward) and 5′-GGCTGTTGTCATACTTCTCATGG-3′ (reverse) for GAPDH.

### Western blotting

Western blot assay was performed following standard procedure. Total protein was extracted using radio immunoprecipitation assay (RIPA) buffer with protease/phosphatase inhibitor cocktail (Roche), measured by BCA protein assay, separated using 8–12% gradient polyacrylamide gel and then transferred onto PVDF membranes. After blocking with 5% BSA, the membranes were incubated with primary antibody at 4 °C overnight and then with secondary antibody at room temperature for 1 h. Bands were detected by FlowChem M, and signal intensities were quantified by Image J.

### Plasmid construction and transfection

The GTSE1 coding sequence was inserted into LV003-IRES-EGFP (Forevergen Biosciences Co., Ltd) and was constructed by GeneCopoeia (Guangzhou, China). Vectors expressing shRNA against GTSE1 (TRCN0000113877) and scrambled shRNA (pLKO.1-NC) were obtained from Sigma. Lentiviruses were produced by co-transfecting constructed plasmids and packaging plasmids psPAX2 and pMD2G into 293 T using Lipofectamine 2000 (Invitrogen, Carlsbad, CA, USA) for approximately 72 h^28^. Culture supernatants were collecteded, filtered, concentrated, and used to infect SMMC-7721 and QGY-7703. After 48 h of infection, infected cells were selected by 2 µg/mL puromycin and confirmed as successfully established by western blotting.

### Colony formation assay

Silencing GTSE1 or control cells (1 × 10^3^) were seeded in each well of 6-well plates and cultured at 37 °C for 14 d in DMEM with 10% FBS. Cells overexpressing GTSE1 or vector (5 × 10^2^) were seeded in 6-well plates for 12 d. Additional culture medium was added to the plates at d 3. Cells were fixed with methanol, stained with 0.5% crystal violet, and dried^29^. The visible clones were counted and the test was repeated three times.

### Cell migration and invasion assay

Migration and invasion assays were performed in 24-well 8-µm pore size transwell plates, with or without pre-coated Matrigel, following the manufacturer’s instructions (Corning, New York, USA). Cells (5 × 10^4^) in DMEM without FBS were seeded into the upper chamber, while the lower chamber was filled with DMEM containing 10% FBS to function as a chemoattractant^30^. The cells were cultivated for 24 h (migration assays) or 48 h (invasion assays). Non-migrating or non-invading cells on the upper side of the chamber were removed by scrubbing. Migrating cells and invasive cells were fixed with methanol and stained with crystal violet. Eight randomly selected images of migrating or invading cells were acquired using an inverted microscope. The experiment was repeated three times.

### Protein degradation

Cycloheximide at a final concentration of 1 mM was added to cells at different time points^31^ (0, 0.5, 1, 1.5, 2, 2.5 h or 0, 8, 16, 24, 32, 40 h). Proteins were then harvested for western blot analysis. The protein degeneration rate was calculated compared with the level of the protein in the control. The protein degradation rates were calculated with the following formula: protein residual percentage after X hours (%) = (protein expression at time X/relative tubulin)/(protein expression at 0 h/relative tubulin) × 100%.

### Cell viability assay by CCK-8

Cells were seeded into 96-well plates at a density of 3 × 10^3^/well. After 24 h, cells were treated with different concentrations of 5-fluorouracil (0, 0.625, 1.25, 2.5, 5, and 10 µM) and cultivated for another 96 h. Relative cell numbers were detected by Cell Counting Kit-8 (CCK8)^32^. Cell viability was calculated by relative absorbance compared to the untreated group (5-fluorouracil concentration at 0 µM) using the following formula: Cell viability (%) = Average (absorbance treated with 5-FU − blank)/Average (absorbance treated without 5-FU − blank) × 100%.

### Statistical analysis

A paired *t*-test was used to analyse the different mRNA levels of GTSE1 in HCC tissues and matched adjacent tissues. Independent *t*-test was applied to analyse differences between two groups. A chi-squared test was employed to analyse the relationship between GTSE1 expression and clinicopathological characteristics. Kaplan-Meier analysis was employed for the survival analysis. All statistical tests were two-sided. Differences with P < 0.05 were considered statistically significant. All statistical tests were performed using SPSS 20.0 statistical software (SPSS Company, Chicago, Illinois, USA).

## Electronic supplementary material

Supplementary
