# Supplementary material for: GTSE1 promotes cell migration and invasion by regulating EMT in hepatocellular carcinoma and is associated with poor prognosis
Source: Sci Rep. 2017 Jul 11;7:5129. doi: 10.1038/s41598-017-05311-2 (PMC5505986; doi:10.1038/s41598-017-05311-2)
Supplement: Supplementary file 1 — Supplementary [file 41598_2017_5311_MOESM1_ESM.pdf]

GTSE1 promotes cell migration and invasion by regulating EMT in hepatocellular carcinoma and is associated with poor prognosis.

Xiaojuan Wu[1,2*], Hongbo Wang[2*], Yifan Lian[2*], Lubiao Chen[1], Lin Gu[2], Jialiang Wang[2], Yanlin Huang[1,2], Meihai Deng[3], Zhiliang Gao[1*], Yuehua Huang[1,2*]

1 Department of Infectious Diseases, The Third Affiliated Hospital of Sun Yat-sen University, Guangzhou, 510630, China.

2 Guangdong Provincial Key Laboratory of Liver Disease Research, The Third Affiliated Hospital of Sun Yat-sen University, Guangzhou, 510630, China.

3 Department of Hepatobiliary Surgery, The Third Affiliated Hospital of Sun Yat-sen University, Guangzhou, 510630, China.

Table of contents

Supplementary Figure 1……………………………………………………………………2

Supplementary Figure 2……………………………………………………………………3

Supplementary Figure 3……………………………………………………………………4

Supplementary Figure 4……………………………………………………………………5

Supplementary Figure 5……………………………………………………………………6

Supplementary Figure 6……………………………………………………………………7

Supplementary Figure 1

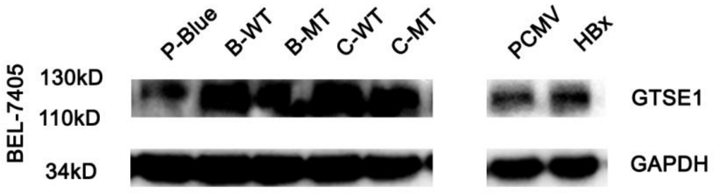

Supplementary Fig. 1. HBV infection elevates GTSE1 expression in BEL-7405.

GTSE1 expression in BEL-7405 treated with different genotype of HBV or HBx or control. PBlue: control; BW: genotype B-wild; BM: genotype B-mutant; CW: genotype C-wild; CM: genotype-C mutant.

Supplementary Figure 2

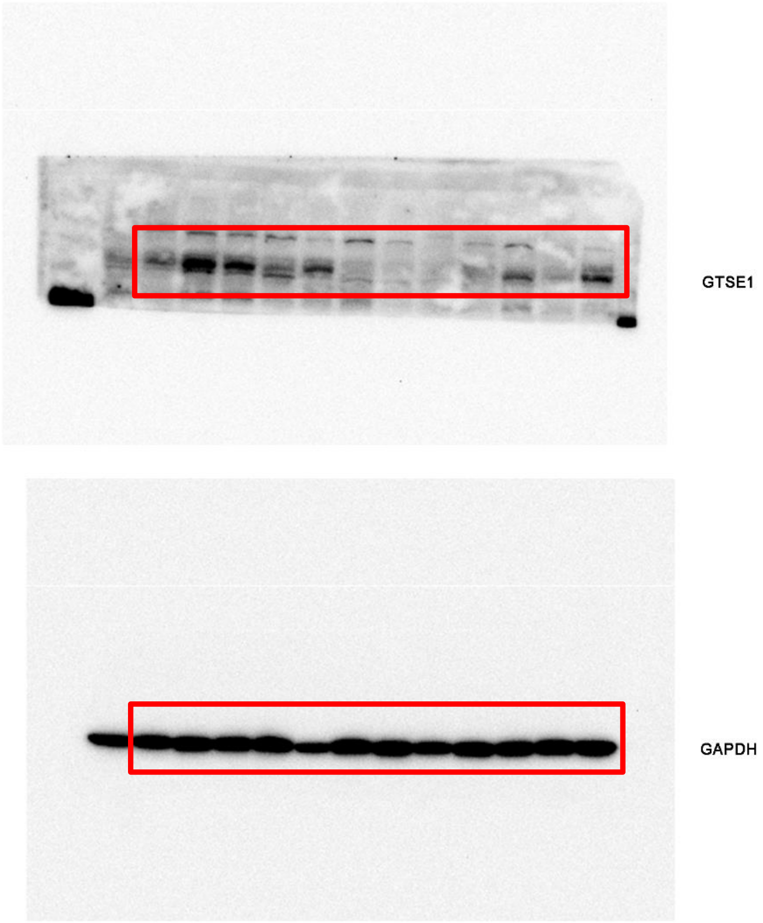

Supplementary Fig. 2. Original data for Fig. 1e

Supplementary Figure 3

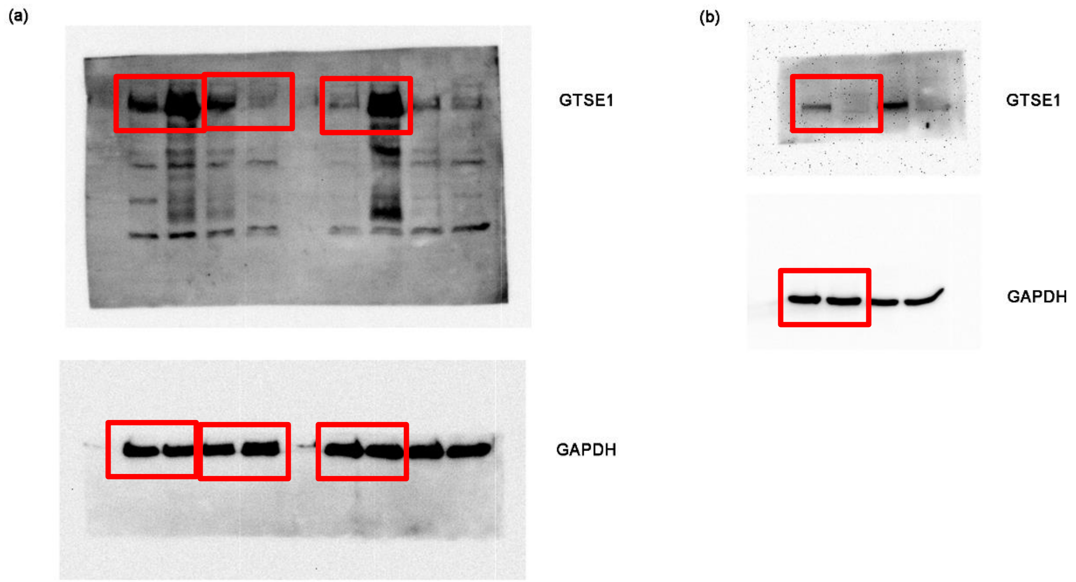

Supplementary Fig. 3. Original data for Fig. 2a

(a) From left to right: QGY-7703 vector, QGY-7703 GTSE1, QGY-7703 shNC, QGY-7703 shGTSE1, SMMC-7721 vector, SMMC-7721 GTSE1.

(b) From left to right: SMMC-7721 shNC, SMMC-7721 shGTSE1.

Supplementary Figure 4

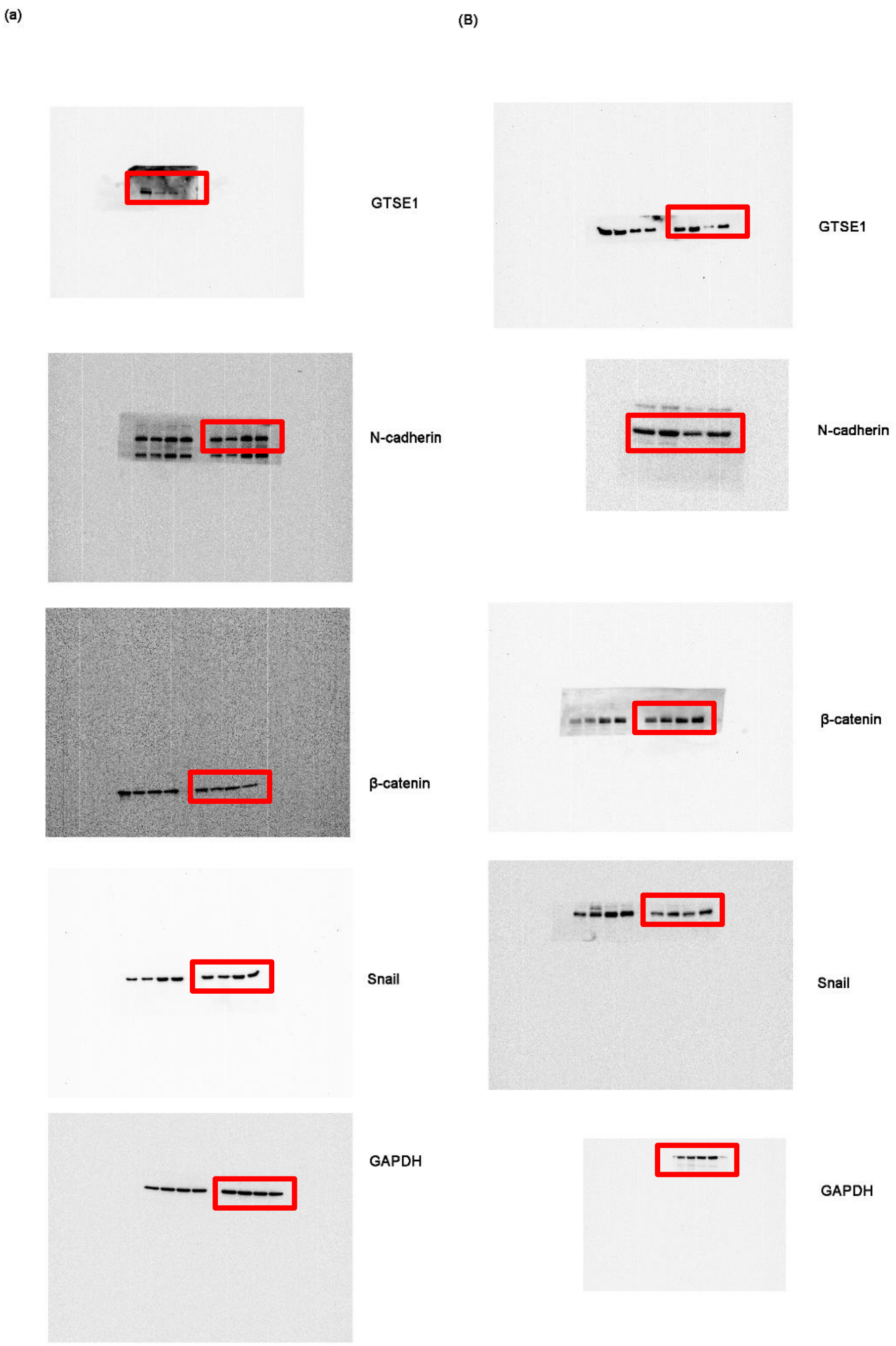

Supplementary Fig. 4. Original data for Fig. 5a

(a) From left to right: QGY-7703 shNC, QGY-7703 shGTSE1, SMMC-7721 shNC, SMMC-7721 shGTSE1.

(b) From left to right: QGY-7703 vector, QGY-7703 GTSE1, SMMC-7721 vector, SMMC-7721 GTSE1.

Supplementary Figure 5

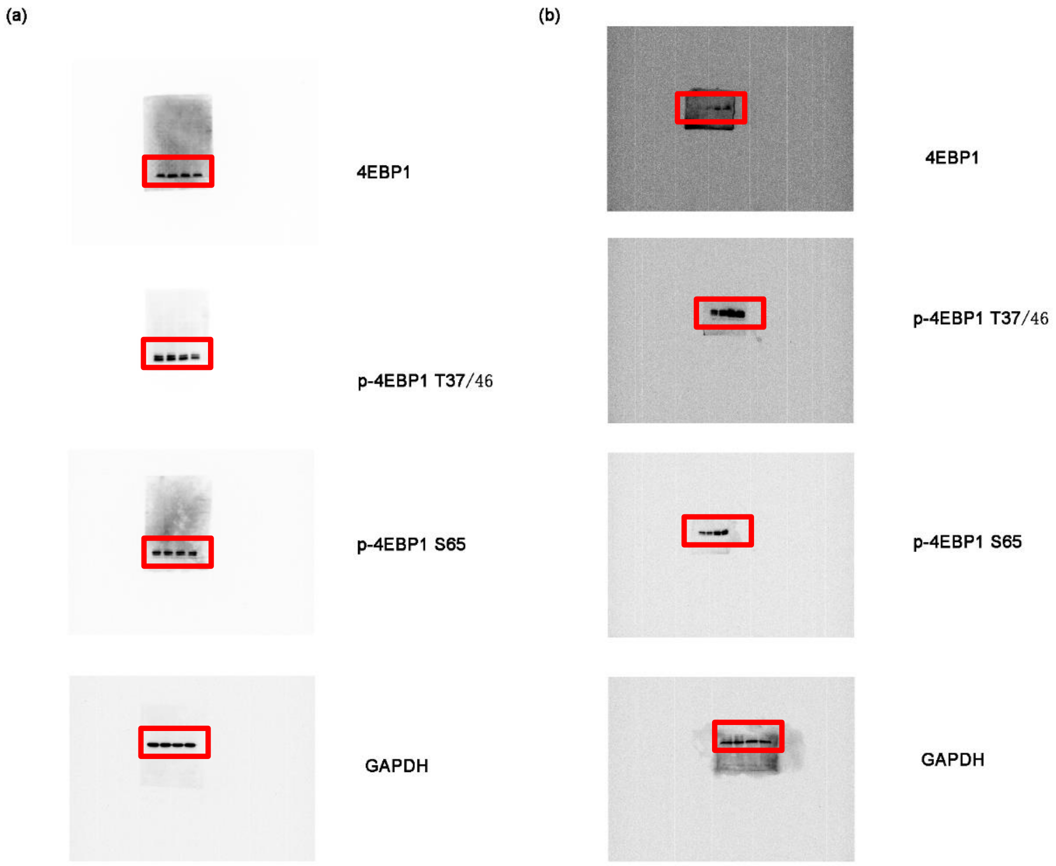

Supplementary Fig. 5. Original data for Fig. 5c

(c) From left to right: QGY-7703 shNC, QGY-7703 shGTSE1, SMMC-7721 shNC, SMMC-7721 shGTSE1.

(d) From left to right: QGY-7703 vector, QGY-7703 GTSE1, SMMC-7721 vector, SMMC-7721 GTSE1.

Supplementary Figure 6

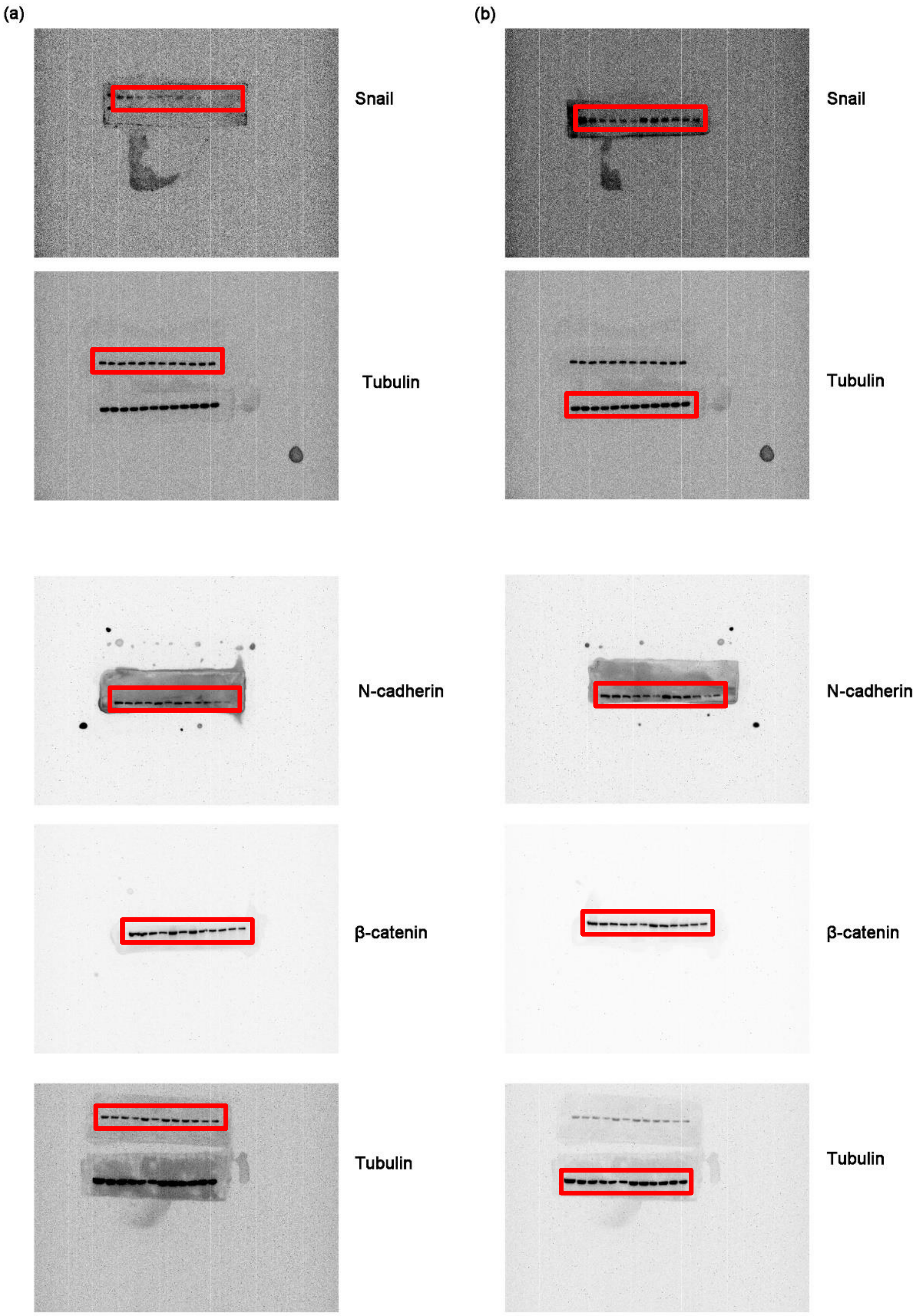

Supplementary Fig. 6. Original data for Fig. 6a
